# Characterisation of the dynamic behaviour of lipid droplets in the early mouse embryo using adaptive harmonic generation microscopy

**DOI:** 10.1186/1471-2121-11-38

**Published:** 2010-06-03

**Authors:** Tomoko Watanabe, Anisha Thayil, Alexander Jesacher, Kate Grieve, Delphine Debarre, Tony Wilson, Martin Booth, Shankar Srinivas

**Affiliations:** 1 Department of Physiology Anatomy and Genetics, University of Oxford, Oxford OX1 3QX, UK; 2 Department of Engineering Science, University of Oxford, Oxford, OX1 3PJ, UK

## Abstract

**Background:**

Lipid droplets (LD) are organelles with an important role in normal metabolism and disease. The lipid content of embryos has a major impact on viability and development. LD in Drosophila embryos and cultured cell lines have been shown to move and fuse in a microtubule dependent manner. Due to limitations in current imaging technology, little is known about the behaviour of LD in the mammalian embryo. Harmonic generation microscopy (HGM) allows one to image LD without the use of exogenous labels. Adaptive optics can be used to correct aberrations that would otherwise degrade the quality and information content of images.

**Results:**

We have built a harmonic generation microscope with adaptive optics to characterise early mouse embryogenesis. At fertilization, LD are small and uniformly distributed, but in the implanting blastocyst, LD are larger and enriched in the invading giant cells of the trophectoderm. Time-lapse studies reveal that LD move continuously and collide but do not fuse, instead forming aggregates that subsequently behave as single units. Using specific inhibitors, we show that the velocity and dynamic behaviour of LD is dependent not only on microtubules as in other systems, but also on microfilaments. We explore the limits within which HGM can be used to study living embryos without compromising viability and make the counterintuitive finding that 16 J of energy delivered continuously over a period of minutes can be less deleterious than an order of magnitude lower energy delivered dis-continuously over a period of hours.

**Conclusions:**

LD in pre-implantation mouse embryos show a previously unappreciated complexity of behaviour that is dependent not only on microtubules, but also microfilaments. Unlike LD in other systems, LD in the mouse embryo do not fuse but form aggregates. This study establishes HGM with adaptive optics as a powerful tool for the study of LD biology and provides insights into the photo-toxic effects of imaging embryos.

## Background

Lipid droplets (LD) are increasingly seen as complex organelles in their own right, and not merely as inert bodies meant simply as energy stores. They are composed of a core of neutral lipids enveloped by a phospholipid monolayer, but also contain a wide variety of proteins, both within the core and embedded in the phospholipid monolayer (reviewed in [1-4]). Fatty acids in LD are used for the generation of energy, membrane synthesis, production of signalling molecules and modification of proteins. Because of this, LD are often found in association with organelles linked to lipid metabolism such as mitochondria, endoplasmic reticulum (ER), endosomes, and peroxiosomes [5]. LD are also involved in several pathological conditions in humans - excess lipid is associated with atherosclerosis [6] and is characteristic of cancer cells [7]. Infection by Dengue or hepatitis C virus leads to an increase in LD number due to the LD having been commandeered for viral particle production [8-10].

LD are dynamic organelles and change shape, volume and location constantly. Evidence from Drosophila embryos and mammalian cell-lines indicates that microtubules are required for directional movement of lipid droplets [11-13]. LD have been reported to coalesce into larger droplets in a microtubule dependent manner [14], though this poses questions regarding the volume/surface area relationship between the constituent droplets and the final LD [2]. Uncertainty remains regarding whether LD actually fuse, as some groups have been unable to observe it occurring [4].

In mammalian embryos, LD are considered primarily to be an energy source, similar to yolk in non-mammalian eggs. However, proteomic approaches in Drosophila suggest that LD might also act as protein-storage organelles in embryos since they contain abundant levels of histones, globular actin, ribosomal subunits and mitochondrial proteins [15]. A similar diversity of proteins has been detected in LD from rat hepatocytes [16]. LD are a dominant feature of pre-implantation embryos from the pig and cow but are also present in mouse and human embryos [17-19]. Studies in mouse embryos have shown an association of LD with organelles such as autophagosomes and mitochondria [20], but in general, little is known about the function and behaviour of LD in mouse embryos.

A major limitation to the study of LD is that most LD dyes work best with fixation, which precludes studying dynamic behaviour. GFP fusion proteins that tag LD have been developed [13,14], but using these in mouse embryos requires the invasive injection of the fusion construct or the production and maintenance of transgenic lines.

Harmonic generation microscopy (HGM) has great potential for the three-dimensional, label-free imaging of developing embryos as demonstrated with zebrafish [21] and Drosophila [22]. The HGM is a laser scanning microscope that takes advantage of second and third harmonic generation (SHG and THG respectively), that result in the generation of photons of half and one-third the illumination wavelength respectively. SHG requires non-centro symmetric media and is generated mainly by structures like muscle, organised microtubules in the mitotic spindle, and collagen fibres [23]. THG probes interfaces and optical inhomogeneities - local variations in the third order non-linear susceptibility (χ^(3)^) and/or refractive index. LD are the major source of THG contrast in cells [22]. HGM has other advantages - emitted photon energy is exactly the same as incident energy so there is no energy deposition in the specimen. It typically employs laser wavelengths in the near infra-red region (900-1500 nm), reducing the absorption and scattering of incident photons, and allowing deeper imaging in thick specimen [24]. HGM is therefore expected to be minimally photo-toxic, making it ideal for studying living embryos.

The performance of microscopes is often compromised by aberrations, arising from imperfections in the optical system or due to physical properties of the specimen. Due to the non-linear dependence of the harmonic signal on the focal intensity, HGM is particularly sensitive to the effects of aberrations. Furthermore, as the illumination wavelengths typically used in HGM are outside the specification of most objective lenses, system aberrations can be significant. The problems caused by aberrations can be overcome using adaptive optics, whereby aberrations are corrected using a dynamic element, such as a deformable mirror [25]. As aberration correction leads to more efficient signal generation, the illumination laser power can be reduced. This is particularly important in reducing phototoxic effects in living specimens, extending the period over which they can be observed.

We have developed an adaptive HGM [26] and new culture techniques for imaging peri-implantation mouse embryos. We find that the THG signal at these stages is generated predominantly by LD. Time lapse HGM shows that LD are very motile, and collide to form larger aggregates that behave as a single unit without actually fusing. This dynamic behaviour is dependent on both microtubules and microfilaments. Finally, we explore the impact of HGM on embryo viability and find that continuous imaging for short time periods does not compromise viability but extended imaging, even with a lower cumulative energy load, does.

## Results

### Adaptive HGM reveals sub-cellular details in the mouse zygote

**Figure 1:**
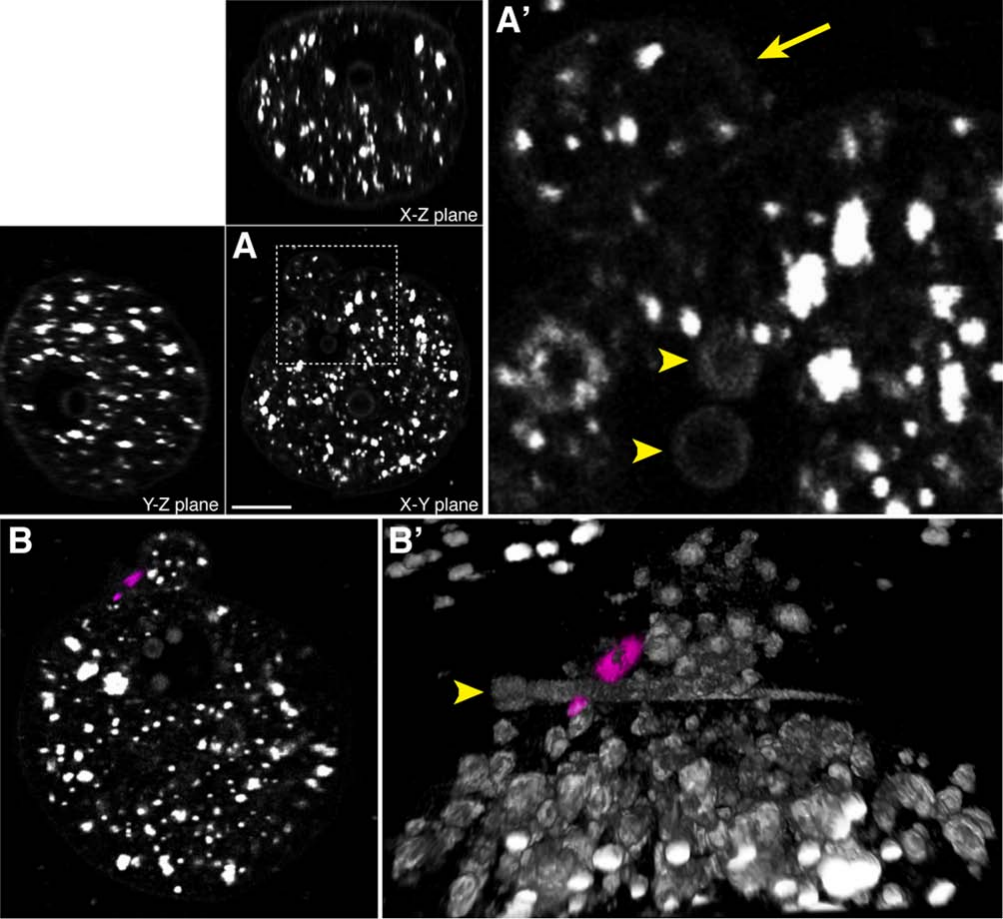
**HGM reveals sub-cellular detail in the living mouse zygote**. (A) THG image volume of a zygote, showing the high-resolution optical sectioning capability of the HGM. Various high-contrast globular structures are visible in the cytoplasm. In addition, the plasma membrane and nucleoli are also visible. (A') high- magnification view of the boxed region in (A) showing details of the nucleolar signal (arrowhead) and the second polar body (arrow). (B) Optical section of SHG and THG signal from a just fertilized embryo. SHG signal (in magenta) is generated by the spindle apparatus of the forming second polar body. (B') Opacity rendering of 3D image data. SHG signal from the spindle is in magenta while THG signal is in grey. A non-fertilizing spermatid is visible outside the zygote (arrowhead). Scale bar in (A) represents 20 μm for (A), 6 μm for (A'), 13.7 μm for (B) and 7.3 μm for (B').

We developed a HGM and culture set-up for maintaining the development of mouse embryos for extended periods while capturing the forward propagated HG signal. We found it essential to incorporate adaptive optics to correct system aberrations [26], in order to acquire data of sufficient quality for detailed analysis (Additional file 1: AO.jpg). Since harmonic generation is a non-linear phenomenon the signal generation is confined to the focus. The resulting optical sections can be used to construct 3D opacity rendered views (Figure. 1A and 1B' and additional files 2 (M1-THG-SHG-sperm.mov), 3 (M2-nucleolus.mov) and 4 (M3-2cell.mov)).

SHG reveals the meiotic spindle of the extruding second polar body (Figure. 1B and 1B' and additional file 2: M1-THG-SHG-sperm.mov). THG reveals many high contrast globular bodies within the zygote. The male and female pronuclei can be discerned as an absence of THG signal within this relatively dense distribution of THG signal. Interestingly, nucleoli within the pro-nuclei can also be clearly seen in THG, though they are relatively faint (Figure. 1A' and additional file 3: M2-nucleolus.mov). THG also reveals structures outside the embryo, such as sperm (Figure. 1B' and additional file 2: M1-THG-SHG-sperm.mov). The plasma membrane is also visible, but is considerably fainter than other features (Figure. 1A).

### THG signal co-localises with lipid droplets

THG signal in mouse embryos has previously been reported to co-localise with membrane bound organelles like mitochondria and ER [27]. However the pattern of THG signal we observe is more reminiscent of LD distribution in other cell types and moreover, THG has been shown to reveal LD with high specificity in a variety of cells from plants, insects and mammals [22]. Therefore, to verify the identity of the high contrast bodies we see by THG, we stained embryos at the 2-cell and morula stage with LipidTox (a neutral lipid dye used for visualising LD), ERtracker (for ER) and mitotracker (for mitochondria) and imaged them simultaneously for THG and two-photon fluorescence (TPF). TPF emission of these dyes in the far-red region of the spectrum was readily obtained in the SHG signal collection channel.

**Figure 2:**
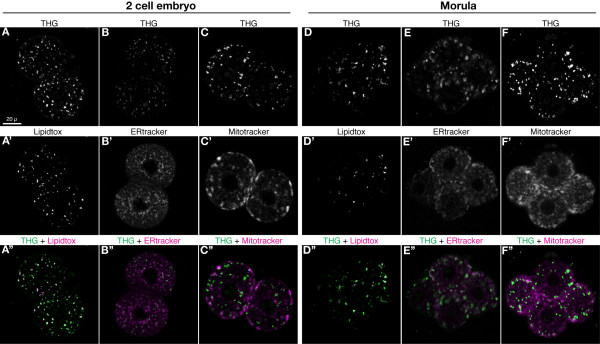
**Colocalization of THG signal with lipid droplets**. (A - A'' and D - D'') Representative 2-cell and morula stage embryos stained with the LD dye LipidTox, showing good colocalization of THG signal with LD. (B - B', C - C', E - E', F - F') 2-cell and morula stage embryos stained with ER and mitochondrial dyes, showing poor colocalization of THG signal with these organelles. The scale bar in (A) = 20 μm and applies to all panels.

We see a high degree of co-localisation of THG and LipidTox signal in both 2-cell embryos and morulae (Figure. 2A and 2D). The Pearson's Correlation coefficients at these two stages are 0.67 and 0.65 respectively (where a coefficient of 1 is perfect correlation and 0 is complete lack of correlation). In contrast, ER (Figure. 2B and 2E) and mitochondria (Figure. 2C and 2F) both show relatively poor co-localisation with THG signal, having Pearson's Correlation coefficients between 0.29-0.39 (0.39 (2-cell) and 0.35 (morula) for ER; 0.28 (2-cell) and 0.37 (morula) for mitochondria). Therefore, on the basis of visual overlap of THG and LipidTox signal, and much higher Pearson's Correlation of 0.65, the high contrast cytoplasmic bodies detected by THG appear predominantly to be LD.

### Dynamic behaviour of embryonic LD

**Figure 3:**
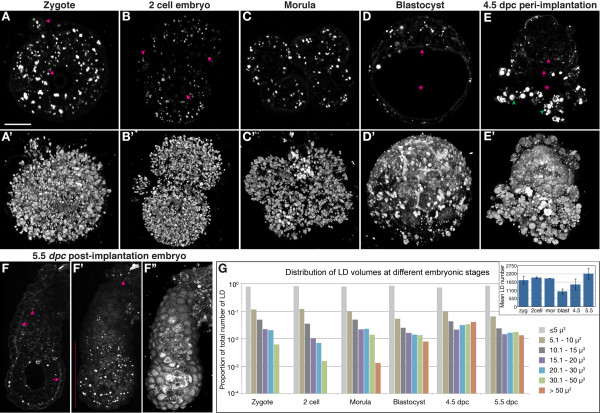
**THG signal changes in a stage and region specific manner**. (A - F) Optical sections. (A' - E' and F'') Opacity rendered 3D views. (F') Extended focus maximum intensity projection. (A - C) Up to the morula stage, THG signal is predominantly from LD which are uniformly distributed throughout the embryo and polar body (arrowheads in A and B). Nucleoli also generate faint THG signal (arrows in A and B). (D) In blastocysts, LD are fewer in number and present in both the ICM (arrow) and TE. Plasma membrane starts to generate more distinct THG signal, visible more clearly in the volume rendering in D'. (E) In the implantation stage embryo, LD are mostly localised to the mural TE (green arrowheads). Plasma membrane is faintly visible and the primitive endoderm (magenta arrow) can be discerned from the epiblast (magenta arrowhead). The blastocoel cavity is marked by an asterisk in (D) and (E). (F) At post-implantation stages, LD are more abundant in the ectoplacental cone (arrowhead in F') and in the distal regions of the egg cylinder (vertical line in F'). Plasma membrane signal becomes more distinct, but is not uniform throughout the embryo, being strongest in the basolateral aspect of the VE (arrows). Nuclei are also now visible, but only in the ExE (arrowhead in F). (G) LD size distribution at different embryonic stages. The distribution shifts towards larger LD as the embryo develops. The inset shows mean LD number at the different stages (n = 4 for all stages except for the 2 cell stage where n = 3). Scale bar in (A) represents 20 μm for (A and C), 30 μm for (B), 32.6 μm for (D), 27.6 μm for (E) and 113.5 μm for (F).

We next characterised LD distribution in embryos at various stages. The zygote, two-cell embryo and eight-cell morula are all roughly similar, densely packed with numerous small LD (Figure. 3A,B,C and additional file 4: M3-2cell.mov). In blastocysts, LD are found in both the inner cell mass (ICM) and trophectoderm (TE). LD appear larger in size, and their number and density appear lower. In the 4.5 days *post coitum *(dpc) peri-implantation embryo, LD are larger than before and are found primarily in the mural TE derived invading giant cells. In 5.5 dpc embryos LD are more abundant in the ectoplacental cone and in the visceral endoderm (VE) overlying the epiblast.

At all stages examined, the majority of LD are less than 5 μm^3 ^in volume (Figure. 3G). However, as the embryo develops from zygote to blastocyst, the distribution of LD volumes shifts to include a greater proportion of larger LD in the range of 5 to 50 μm^3^. LD larger than 50 μm^3 ^are only seen in the late morula and beyond and show a distinct increase at the blastocyst stage, concomitant with a drop in the average number of LD (inset of Figure. 3G). After implantation (4.5 dpc), the number of LD increases again, though the distribution of sizes remains similar to that seen in blastocysts.

**Figure 4:**
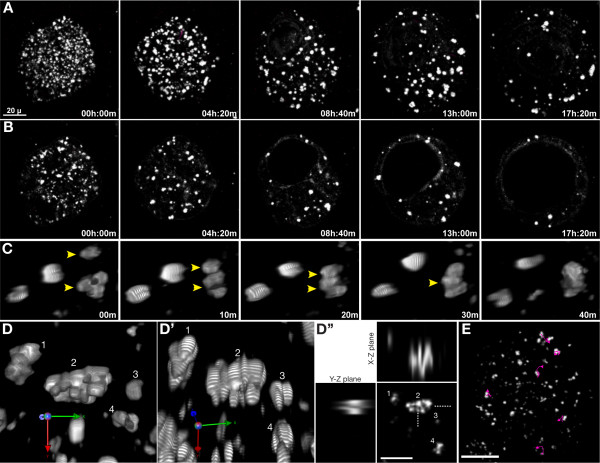
**LD show dynamic behaviour during development**. (A) Extended focus projections and (B) optical sections of an embryo imaged from the morula to blastocyst stage. LD move actively within the embryo in the course of development, and gradually become fewer in number and larger. (C) 3D Opacity rendering of LD showing them merging to form larger LD. (D, D') Two different 3D views of several LD, showing that larger LD appear to be aggregates of several smaller LD. Corresponding LD are numbered in the two views, and the orientation is shown at bottom left. One can see that LD aggregates appear irregular because they are composed of several bodies. Banding and stretching of LD along the Z axis in (D') are rendering artefacts. (D'') Optical section of the LD in (D), showing that larger LD are aggregates of smaller LD. LD numbers correspond to those in (D). (E) Tracks of representative LD (magenta lines) over a one hour period. LD do not appear to move in a coordinated manner across the embryo. Their tracks are convoluted to varying extents. Scale bar in (A) = 20 μm and applies to (A and B). Scale bar in (D'') = 10 μm and in (E) = 20 μm

To test if the large LD in blastocysts are formed by fusion of existing LD, we imaged morulae for up to 24 hours (up to the blastocyst stage) at 10 min time intervals. LD show dynamic behaviour in the course of development, moving continuously (Figure. 4A - C,E and additional files 5 (M4-emb-proj.mov) and 6 (M5-emb-sect.mov)). They frequently collide and merge (Figure. 4C), but do not actually fuse, and instead form aggregates. Larger LD are irregular in outline and often appear to be comprised of several LD very closely apposed to one another (Figure. 4D and 4D'). Optical sections through LD at this stage also indicate that they are associations of several distinct bodies (Figure. 4D''). Once formed, these aggregates move together as a single unit, though their components appear to 'jostle' one another as they move (Additional file 7: M6-aggregate.mov). None of these aggregates underwent fusion of their components, and instead persisted till the end of the time-lapse experiment (for up to 8 hours, depending on when the aggregate formed). To confirm that LD aggregates are not artefacts of THG imaging, we verified their existence using standard confocal microscopy of LipidTox stained embryos (Additional file 8: LipidToxAggregate.jpg).

Tracks of LD are convoluted (Figure. 4E) to varying degrees, with larger LD in forming blastocysts having more linear paths. LD in different regions of the embryo do not appear to move in a coordinated manner or direction (Figure. 4E). LD have an estimated velocity of 233 nm/min (SD = 46 nm/min). In the human hepatoma cell line HuH-7, two distinct types of LD movement have been reported - the majority oscillate in place, but a small number show occasional rapid directional movement at up to 2.5 μm/sec [13]. To capture any such rapid movement, we imaged 6 embryos at a single optical plane at 1 second intervals for up to 5 min, but did not see any fast moving LD (Additional file 9: M7-emb-1sec.mov).

### LD behaviour is actin and microtubule dependant

**Figure 5:**
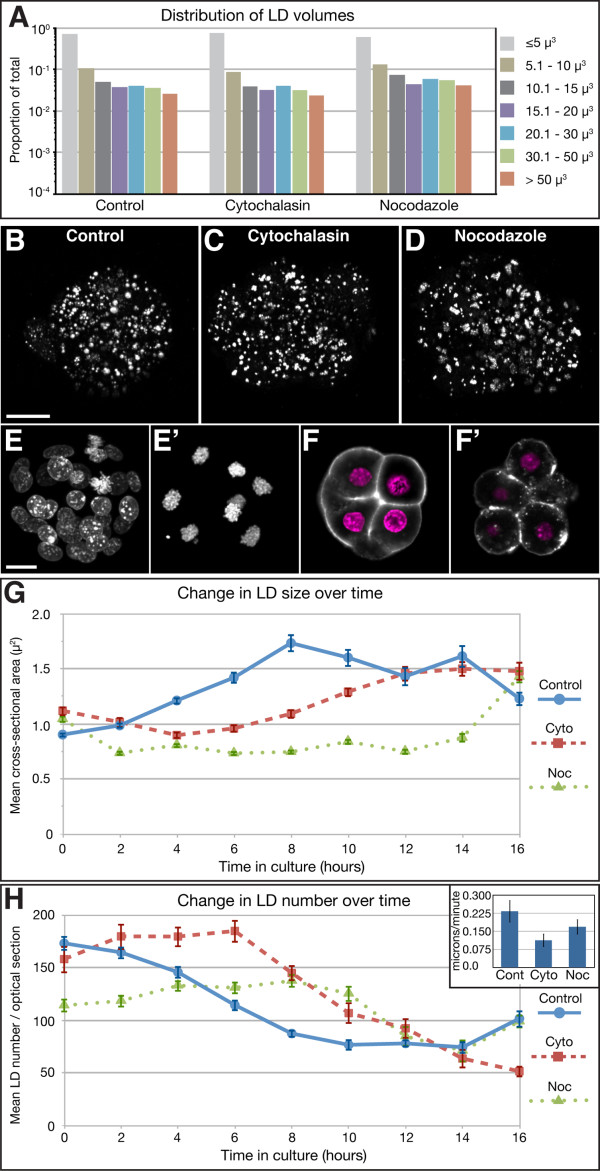
**Dynamic behaviour of LD is both microtubule and microfilament dependent**. (A) LD size distribution in embryos after culture for 22 hours in cytochalasin D and nocodazol. Control embryos were cultured in DMSO (carrier). There is no difference in the distribution of LD sizes (n = 4 embryo for each condition). (B, C, D) Extended focus projections of THG signal from representative control, Cytochalasin D and Nocodazole treated embryos, showing no obvious difference in LD. (E, E') Extended focus projections of confocal scans of fluorescence from DAPI stained nuclei (magenta) in control (E) and nocodazole (E') treated embryos. As expected, nuclei in the later are arrested at the metaphase stage, as seen by the condensed chromosomes. (F, F') Optical confocal section of fluorescence from F-actin stained with Phalloidin (grey) and nuclei stained with DAPI (magenta), in control and cytochalasin D treated embryos. As expected, F-actin localisation is disrupted in the latter. (G, H) Change in average LD size and number over time in embryos cultured in the presence of cytochalasin D and nocodazole (n = 12 sections from 4 embryos for each condition). Both inhibitors cause a clear delay in the formation of larger LD. The inset in (H) shows the estimated average velocity of LD. Treatment with the two inhibitors causes a significant reduction to LD velocity as compared to control LD (p < 0.0001, Students T-test). Scale bar in (B) = 20 μm and applies to (B, C and D). Scale bar in (E) = 20 μm and applies to (E, E', F and F')

To investigate the mechanism underlying LD movement in the mouse embryo, we cultured morulae overnight in the presence of either nocodazole or cytochalasin D, that interfere with the polymerisation of microtubules and microfilaments respectively. As expected, both nocodazole and cytochalasin D treated embryos failed to form blastocysts and the latter underwent decompaction [28-30]. However there was no difference in the distribution of LD sizes in either nocodazole or cytochalasin D treated embryos when compared to control (DMSO) treated embryos (Figure. 5A). The LD in such embryos also look grossly similar to those in control embryos (Figure. 5B-D). We verified the activity of nocodazole by visualising chromosomes, that arrest at metaphase as expected [28] (Figure. 5E,E' and additional file 10: M8-noc-control.mov). We verified cytochalasin D activity by visualising F-actin, which shows the expected abnormal distribution [31] (Figure. 5F,F').

Next, we cultured morulae overnight in the presence of inhibitors while imaging them at 10 min intervals. In the presence of both inhibitors, LD move and form aggregates (Additional files 11 (M9-cyto.mov) and 12 (M10-noc.mov)). However, their estimated average velocity is significantly lower, 169 nm/min (SD 37 nm/min) and 113 nm/min (SD 27 nm/min) respectively in nocodazole and cytochalasin D (p < 0.0001 for both compared to control, Student's T-test) (inset Figure. 5H). Calculations of the average LD cross sectional area showed an increase in LD size in controls, accompanied by a reduction in LD number (Figure. 5G,H). There is a noticeable lag in the formation of larger LD both in the presence of nocodazole and cytochalasin D, the delay being more marked with the former (Figure. 5G,H). Interestingly, in the presence of either inhibitor, LD were able to recover and attain the normal average size by 16 hours in culture (Figure. 5G,H and additional files 11 (M9-cyto.mov) and 12 (M10-noc.mov)).

### HGM can compromise the viability of developing embryos

Morulae typically form blastocysts when imaged over-night with the HGM and can even hatch out of the zona as normal (Table 1 and additional files 5 (M4-emb-proj.mov) and 6 (M5-emb-sect.mov)). This is comparable to the development of embryos in tissue-culture incubators and suggests that the cellular machinery for morula compaction, blastocyst formation and hatching are not affected by the pulsed laser illumination used.

**Table 1:** Viability of embryos imaged with HGM

**Number of embryos**	**Stage at start of experiment**	**Duration**	**Stage at end of experiment**	**Time interval between imaging (min)**	**Z levels imaged**	**Estimated total energy load per embryo**	**Number developing normally**
10	Morula	22 hours	Blastocyst	20	7	4 J	0
12	Morula	22 hours	Blastocyst	20	7	2.5 J	0
8	Morula	22 hours	Blastocyst	60	11	0.9 J	1
8	Morula	24 hours	Blastocyst	not imaged	not imaged	~ 0 J	6
9	Zygote	10 min	Zygote	continuous	10	16 J	6

Embryos were subject to different imaging conditions and their development assessed. From the recipient with embryos subject to 0.9 J of energy, in addition to 1 normal embryo, 3 empty decidua and 2 resorbing embryos were found.

To test the viability of embryos imaged overnight, we transferred them into pseudo-pregnant recipients to see if they developed further. 10 morulae subject to 4 J over 22 hours developed into blastocysts but failed to yield any fetuses at 18.5 dpc, one day before parturition (Table 1). 12 morulae subject to a reduced energy load of 2.5 J also failed to yield any fetuses at 18.5 dpc. To determine when imaged embryos might be dying, we subjected 8 embryos to 0.9 J of energy over 22 hours and dissected them out at 8.5 dpc. 1 normal, live and 2 dead, resorbing embryos were found, in addition to 3 empty uterine decidua. This suggests some of the embryos imaged at this energy level implanted but only some of those developed past gastrulation and then started to die shortly after (Table 1). 8 control embryos cultured in the microscope incubator for 24 hours without imaging yielded 6 normal, live fetuses, in line with the normal recovery rate for our group.

Embryos imaged with HGM have been reported to give rise to healthy live-born pups when exposed to 10 min of continuous illumination, estimated at 27 J of energy [27]. To reconcile this result with our own that suggests that HGM imaging severely compromises viability, we tested the effect of a single extended dose of laser illumination. 9 zygotes were exposed to 10 min of continuous laser illumination, equivalent to a cumulative energy load of 16 J and then directly transferred into a recipient. 6 normal live fetuses were obtained at 8.5 dpc, which is within the survival range obtained routinely by our group with transfers of non-manipulated embryos (Table 1).

## Discussion

### Adaptive HGM for high resolution imaging of LD

By combining HGM with adaptive optics, we have compensated for system induced aberrations, enabling us to acquire images with improved resolution and signal levels at relatively low laser powers. This has facilitated a high-resolution characterisation of the mouse embryo by HGM, revealing features like the spindle, LD, nucleoli and sperm.

The high contrast THG signal in pre-implantation embryos is predominantly from LD. The relatively low co-localisation with mitochondria and ER shows that THG is not inevitably generated by all membrane bound organelles. Furthermore, nucleoli, centres of active rRNA transcription not membrane bound or particularly rich in lipids also generate faint THG at early stages. The limited correlation of THG with ER and mitochondria might be the result of low levels of THG generated by these organelles. Arguing against this however is the observation that mitochondria show a characteristic accumulation to regions of cell-cell contact during embryonic cleavage divisions [32,33] which is not observed with THG. A more likely explanation seems to be the close association of LD with mitochondria and ER [13,34,35,5].

In the implanting 4.5 dpc embryo, LD are most abundant in the mural TE derived invading giant cells as compared to other regions of the embryo. Embryonic lipid has been implicated in the activation of uterine anadamide hydrolase during implantation [36], required for the inactivation of anadamide which otherwise impairs embryo development. Thus it is possible that the large LD in the invading giant cells have a role in implantation. By 5.5 dpc, LD are localised mostly to the ectoplacental cone and VE. Within the VE, LD are more abundant in the distal regions, consistent with regional differences in the VE at this stage [37].

The specificity of THG to LD in embryos makes it potentially useful in assisted reproductive technology (ART), in monitoring the quality of embryos. Elevated LD content has been associated with suboptimal embryo health and increased cryo-sensitivity in bovine embryos [38]. In porcine embryos, in vitro cultured embryos had more LD than non-cultured (presumably healthier) embryos [39]. THG can be used to quantify LD volume and number in live embryos without staining, providing objective criteria for assessing embryo quality. The culture set-up reported here can also be used to maintain cell lines imaged with HGM, making it a useful tool for visually probing lipid dynamics in cell-based models of pathological conditions like cancer, diabetes and obesity.

### Dynamic behaviour of LD is both microtubule and microfilament dependent

High resolution time-lapse recordings show that LD grow in size by forming aggregates. Experiments in tissue-culture cells loaded with lipids suggest LD fuse on the time-scale of minutes [14]. In the mouse embryo however, individual LD do not actually fuse, but remain as clusters of associated LD for several hours. LD aggregates behave as single units, suggesting the individual droplets are actively held together. It is possible that these LD aggregates eventually fuse or conversely, disaggregate over a period of days, since LD in 4.5 dpc and 5.5 dpc embryos are more spherical in outline. Current limitations in culture technology preclude these possibilities being directly investigated.

Our results do not preclude other mechanisms for the increase in size of LD, such as the continuous deposition of neutral lipids or *de novo *generation of large LD. Indeed, after a dip at the blastocyst stage, the average number of LD increases again post-implantation (4.5 dpc and 5.5 dpc), though the distribution of LD sizes remains roughly the same, suggesting that new LD are being produced. This *de novo *generation of LD occurs shortly following implantation, consistent with a maternal source for the material needed to produce the new LD.

Studies on LD movement have focused on the role of microtubules and associated motors. We show here an additional, previously unreported dependence of LD movement on the microfilament cytoskeleton. This is consistent with reports of actin filaments within LD, detected by electron microscopy and biochemical analyses [16,40,15]. A dynamic meshwork of actin filaments in the mouse oocyte has recently been shown to be responsible for the movement and positioning of the meiotic spindle [41]. It is possible such a mesh-work contributes to LD movement - time lapse movies of this actin network [41] are reminiscent of the 'jiggling' movement of LD seen in our 1 second time-interval recordings (Additional file 9: M7-emb-1sec.mov). As in Drosophila embryos and cultured mammalian cell lines, LD movement and aggregation in mouse embryos is also microtubule dependent. Disruption of microfilaments causes a greater reduction in the average velocity of LD than disruption of microtubules. However neither is absolutely required for LD movement since within 16 hours of culture, embryos in both inhibitors regain normal LD size and number. This is similar to results in Drosophila oocytes, where mutants of the microtubule component α4-tubulin show a reduction in LD velocity and overall dynamic behaviour, but not a complete arrest [12]. Our results therefore suggest that neither cytoskeletal component is exclusively responsible for mediating LD movement and aggregation, but possibly act in concert with other mechanisms in the cell.

### Long term harmonic generation imaging compromises embryo viability

Embryos imaged from the morula to blastocyst stage (subject to a cumulative energy load ranging from 0.9 J to 4 J) appear to develop entirely normally on the basis of morphological criteria. However, when transferred into recipients, they consistently fail to develop further. This implies that imaged embryos, though apparently normal during the course of imaging, have accumulated photo-damage that severely compromises their later development. This highlights the importance of assessing the impact of imaging on the viability of embryos not merely be seeing if they develop normally *in vitro*, but by determining if they can develop beyond gastrulation when transferred into recipients.

Curiously, zygotes imaged continuously for 10 min (subject to 16 J of cumulative energy load) do not suffer impaired viability when transferred into recipients. Similar results were obtained by Hsieh et al. [27]. These results are surprising because they suggest that small doses of energy provided over an extended period can be more damaging than even an order of magnitude larger cumulative dose provided continuous over a short duration. One possible explanation for this is that embryonic blastomeres are particularly sensitive to photo-damage at specific transient points in the cell cycle (such as the M phase for example) and insensitive at other times, so imaging them repeatedly over an extended period increases the probability of catching them during a sensitive phase. Optical absorption coefficients of the principal tissue components in the embryo are reported to be relatively low at the 1200 nm wavelength range [24] and harmonic generation does not require absorption of photons, so theoretically embryos should not suffer photo-damage at all. Therefore, it is likely that phototoxic effects arise through multi-photon absorption (equivalent to linear absorption at 615 nm or 410 nm) rather than at the illumination wavelength of 1235 nm.

## Conclusions

HGM with adaptive optics enables one to capture high resolution 4D image data of early mouse embryos. The THG signal generated by pre-implantation embryos is predominantly from LD rather than organelles such as ER or mitochondria. LD are larger in peri-implantation embryos than in morula and earlier stage embryos. LD in pre-implantation embryos show a previously unappreciated complexity of behaviour, moving continuously and apparently without overall coordination. This movement leads them to collide and form aggregates but not fuse, in contrast to the reported behaviour of LD in other systems. The dynamic behaviour of LD is dependent not only on microtubules, but also microfilaments. Photo-toxicity from long term HHG imaging is far from negligible and should remain a major consideration in its use for applications such as ART. However, embryos can be imaged for up to 10 min continuously without any apparent adverse effects, a duration more than sufficient to capture a high resolution 3D image volume, especially in combination with adaptive optics to reduce illumination intensities without sacrificing image quality. HGM therefore still retains promise as an attractive and viable tool in examining living mammalian embryos, in addition to being a powerful means of studying LD dynamics in cultured cell lines used to model pathological conditions like cancer, diabetes and atherosclerosis.

## Methods

### Mouse husbandry and embryo collection

Mice were housed in a 12 hour dark, 12 hour light cycle. CD1 females were crossed with CD1 males to obtain stage specific embryos. Noon of the day of finding the mating plug was designated 0.5 dpc. Embryos were dissected in M2 medium (Sigma M7167). All experimental procedures complied with Home Office regulations and were approved by a local Ethical Review Committee.

### Adaptive harmonic generation microscopy

A laser scanning HGM suitable for long-term imaging of cultured mammalian embryo was constructed [26]. The entire microscope was enclosed by a chamber to maintain it at 37°C. The objective and sample were enclosed by a small plastic chamber into which a humidified mixture of 5% CO2 in air could be supplied. For both single stage as well as time-lapse imaging, embryos were placed in a drop of medium in a glass bottom dish (Mattek). The drop of medium was covered with a coverglass supported by Mylar spacers of 200 μm thickness. The dish was filled with embryo-grade mineral oil (Sigma M5310) to prevent evaporation of the medium.

Embryos were imaged using a chromium forsterite laser (Mavericks, Del Mar Photonics) emitting 65 fs pulses at 120 MHz repetition rate, central wavelength 1235 nm and output average power around 200 mW. A deformable membrane mirror (MIRAO 52-e, Imagine Eyes) was incorporated into the microscope to correct system aberrations [26]. A pair of galvanometric mirrors was used for in plane laser scanning. A piezo actuator attached to the stage enabled axial scanning of the specimen. A 40x, NA = 1.15 objective lens (Olympus UApo/340 water immersion) was used to focus the excitation beam. Harmonic generation signal was collected in trans-configuration by an oil immersion condenser (NA = 1.4). The SHG and THG were separated by a dichroic filter and detected simultaneously using two photomultiplier tubes. Unless otherwise stated, the average laser power at the sample was approximately 35 mW with a pixel dwell time of 8 μsec.

For co-localisation studies, LD were imaged using THG and stained organelles (ERtracker, Mitotracker, LipidTox) were imaged though TPF excitation using the same laser illumination.

### Embryo imaging and culture

For single stage imaging, embryos were imaged in M2 medium at 37°C in ambient air, at a typical in-plane pixel size of 0.2 × 0.2 to 0.3 × 0.3 μm^2 ^and axial step-size of 0.5-1.0 μm. At least 5 embryos were imaged for each stage. For time-lapse experiments, embryos were imaged in KSOM (Millipore MR-050P-5F) supplemented with Essential Amino Acids (Invitrogen 11130-036), Non-Essential Amino Acids (Invitrogen 11140-350) and sodium pyruvate at 37°C in a 5%CO2/air mix, at a typical in-plane pixel size of 0.2-0.3 μm and axial step-size of 1.0-3.0 μm. Depending on the experiment, embryos were imaged for various durations (from 5 min to 24 hours) and at various time-intervals (continuously to 1 hour).

For inhibitor studies, embryos were cultured in the presence of 5 μg/ml nocodazole (stock 5 mg/ml in DMSO), 1 μg/ml cytochalasin D (stock 1 mg/ml in DMSO) or DMSO as a carrier control (1:1000 dilution) in culture medium.

### Staining

We stained a minimum of 6 embryos at each stage with each dye. ERtracker Red (Invitrogen E34250, 1:1000 dilution), Mitotracker DeepRed (Invitrogen M22426, 1:2000 dilution) and HCS LipidTox DeepRed (Invitrogen H34477, 1:200 dilution) were used to stain the ER, mitochondria and LD respectively. All staining was done without fixation. Mitotracker and LipidTox staining was done in KSOM+AA, while ERtracker was in HBSS (Sigma), all incubated in 5% CO2 at 37°C for 30-45 min, washed briefly 3 times in M2 medium at room temperature and then imaged in M2 at 37°C.

### Image analysis and Quantification

Image analysis, 3D rendering, calculation of LD size and number, LD tracking and computation of Pearson correlation coefficients [42] were done using Volocity software (Improvision). LD velocity was estimated by tracking 16 LD from 4 morula for each condition over a period of at least an hour and dividing the track length by the time. LD size in the inhibitor time lapse experiment was calculated as the mean cross sectional area of all the LD in the three central optical sections of four embryos, for each condition.

## Authors' contributions

TWa carried out the embryo isolation, staining, inhibitor studies, LD quantitation and drafted the manuscript. AJ, AT and KG built the HGM with input from DD and MB. AT and AJ performed the imaging of embryos. SS, MB and TWi conceived of the study. SS coordinated the study and drafted the manuscript. All authors read and approved the final manuscript.

## Supplementary Material

Additional file 1 **Aberration correction improves HGM image quality**. (A) Schematic of the adaptive harmonic generation microscope. Lx, lens; Mx, mirror; BSx, beam splitter; DM, deformable mirror; O, objective; S, specimen; C, condenser; D, dichroic; PMTx, photomultiplier tubes for THG (blue) and SHG/TPF (green) signal detection. He-Ne laser (dashed outline) is used for DM characterization and this path is disabled during imaging. (B) Representative THG image of 5.5 dpc mouse embryo, a region of size 30 μm × 30 μm × 15 μm, approximately 90 μm deep in the sample before aberration correction (C) after correcting system induced aberrations. Scale bar is 10 μm. (D) The correction phase function applied to the DM. This consists mainly of spherical aberration, probably due to incomplete coverglass thickness compensation in the objective lens. Compensating system aberrations results in an over all signal improvement of nearly 40%. Click here for file

Additional file 2 **SHG and THG image of mouse zygote**. Opacity rendering of SHG (magenta) and THG (grey) image volumes of the surface of a mouse zygote. The meiotic spindle of the second polar body is visible in SHG. THG reveals LD and sperm. Click here for file

Additional file 3 **THG image of nucleolus**. Opacity rendering of a THG image volume of LD and a nucleolus. The spherical nucleolus is within the nucleus, that is marked by an absence of THG signal. Click here for file

Additional file 4 **THG image of 2-cell mouse embryos**. Opacity rendering of a THG image volume of densely packed LD in a two cell embryo. Click here for file

Additional file 5 **Time-lapse of embryo development: extended focus projection**. Extended focus projection of a mouse embryo imaged with THG (grey) and SHG (magenta). Imaging was started at the compacted morula stage and development followed till the blastocyst stage. Images were captured at 20 minute intervals. The majority of THG signal is generated by lipid droplets, which become fewer and larger over the course of development. Use keyboard arrow keys to navigate frame by frame through the animation. Scale = 20 μm. Click here for file

Additional file 6 **Time-lapse of embryo development: optical section**. Optical sections of the mouse embryo in additional file 5 (M4-emb-proj.mov), imaged with THG (grey) and SHG (magenta). Imaging was started at the compacted morula stage and development followed till the blastocyst stage. Images were captured at 20 minute intervals. The majority of THG signal is generated by lipid droplets, which become fewer and larger over the course of development. Use keyboard arrow keys to navigate frame by frame through the animation. Scale = 20 μm. Click here for file

Additional file 7 **Time-lapse of LD aggregate moving and changing shape**. Images were captured at 10 minute intervals. LD aggregates move as single units while continuously changing shape. The top panel is an opacity volume rendering while the bottom panel shows optical sections in the image plane (X-Y plane) and X-Z and Y-Z projections. At the centre of the opacity rendering is the LD that is followed in the optical sections. One can see the LD aggregate continuously changing shape while moving, and the component LD appearing to 'jostle' each other. LD aggregates persist and behave as single units for over 7 hours. Use keyboard arrow keys to navigate frame by frame through the animation. Scale = 5 μm. Click here for file

Additional file 8 **LD aggregates stained with LipidTox**. Two examples of LD aggregates from a live blastocyst stained with LipidTox and imaged by confocal microscopy. (A' and B') are different 3D views of the LD in (A and B) respectively. Corresponding LD are numbered in the two views, and the orientation is shown at bottom left. (A'' and B'') Optical section of the LD in (A and B) respectively, showing that LD are aggregates of smaller LD. LD numbers correspond to those in the volume renderings. Scale bar in (A'') = 2 μm and in (B'') = 5 μm. Click here for file

Additional file 9 **Time-lapse of LD in embryo at 1 second interval - change movie**. Optical section of a mouse compacted morula imaged with THG at 1 second interval. The high-contrast lipid droplets do not move appreciably, but appear to jiggle in place. Use keyboard arrow keys to navigate frame by frame through the animation. Scale = 20 μm. Click here for file

Additional file 10 **Nuclear stain of control and Nocodazole treated embryos**. Opacity renderings of morulae cultured in DMSO (carrier control) and Nocodazole overnight. Embryos were fixed and stained with DAPI to visualise DNA. The control morula (at left) developed normally, and underwent several rounds of division. One can see interphase nuclei as regular spheroids and one cell in metaphase towards the top right of the embryo. In contrast, the nocodazole treated embryo suffered metaphase arrest, as evident from their bumpy appearance due to condensed chromosomes and the presence of only 8 staining bodies. Click here for file

Additional file 11 **Time-lapse of embryo development in cytochalasin D change movie**. Extended focus projection of THG signal from a mouse morula cultured in the presence of cytochalasin. Images were captured at 10 minute intervals. The embryo undergoes decompaction as a result of cytochalasin treatment. LD continue to move actively and aggregate into larger LD. Use keyboard arrow keys to navigate frame by frame through the animation. Scale = 20 μm. Click here for file

Additional file 12 **Time-lapse of embryo development in nocodazole change movie**. Optical section of THG signal from a mouse morula cultured in the presence of nocodazole. Images were captured at 10 minute intervals. The embryo fails to form a blastocoel cavity as a result of nocodazole treatment. LD continue to move actively and aggregate into larger LD. Use keyboard arrow keys to navigate frame by frame through the animation. Scale = 20 μm. Click here for file
