# Supplementary figures and images for: Characterisation of the dynamic behaviour of lipid droplets in the early mouse embryo using adaptive harmonic generation microscopy
Source: BMC Cell Biol. 2010 Jun 3;11:38. doi: 10.1186/1471-2121-11-38 (PMC3238212; doi:10.1186/1471-2121-11-38)

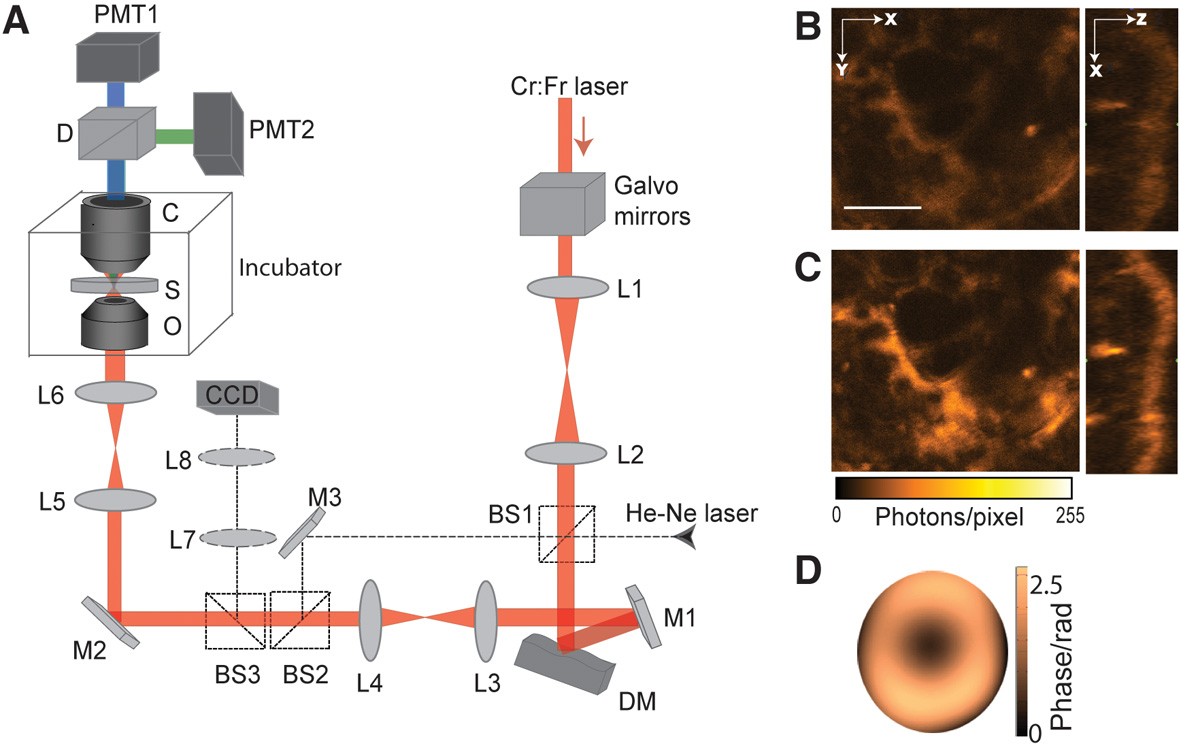

Supplement: Additional file 1 — Aberration correction improves HGM image quality. (A) Schematic of the adaptive harmonic generation microscope. Lx, lens; Mx, mirror; BSx, beam splitter; DM, deformable mirror; O, objective; S, specimen; C, condenser; D, dichroic; PMTx, photomultiplier tubes for THG (blue) and SHG/TPF (green) signal detection. He-Ne laser (dashed outline) is used for DM characterization and this path is disabled during imaging. (B) Representative THG image of 5.5 dpc mouse embryo, a region of size 30 μm × 30 μm × 15 μm, approximately 90 μm deep in the sample before aberration correction (C) after correcting system induced aberrations. Scale bar is 10 μm. (D) The correction phase function applied to the DM. This consists mainly of spherical aberration, probably due to incomplete coverglass thickness compensation in the objective lens. Compensating system aberrations results in an over all signal improvement of nearly 40%. [file 1471-2121-11-38-S1.jpeg]

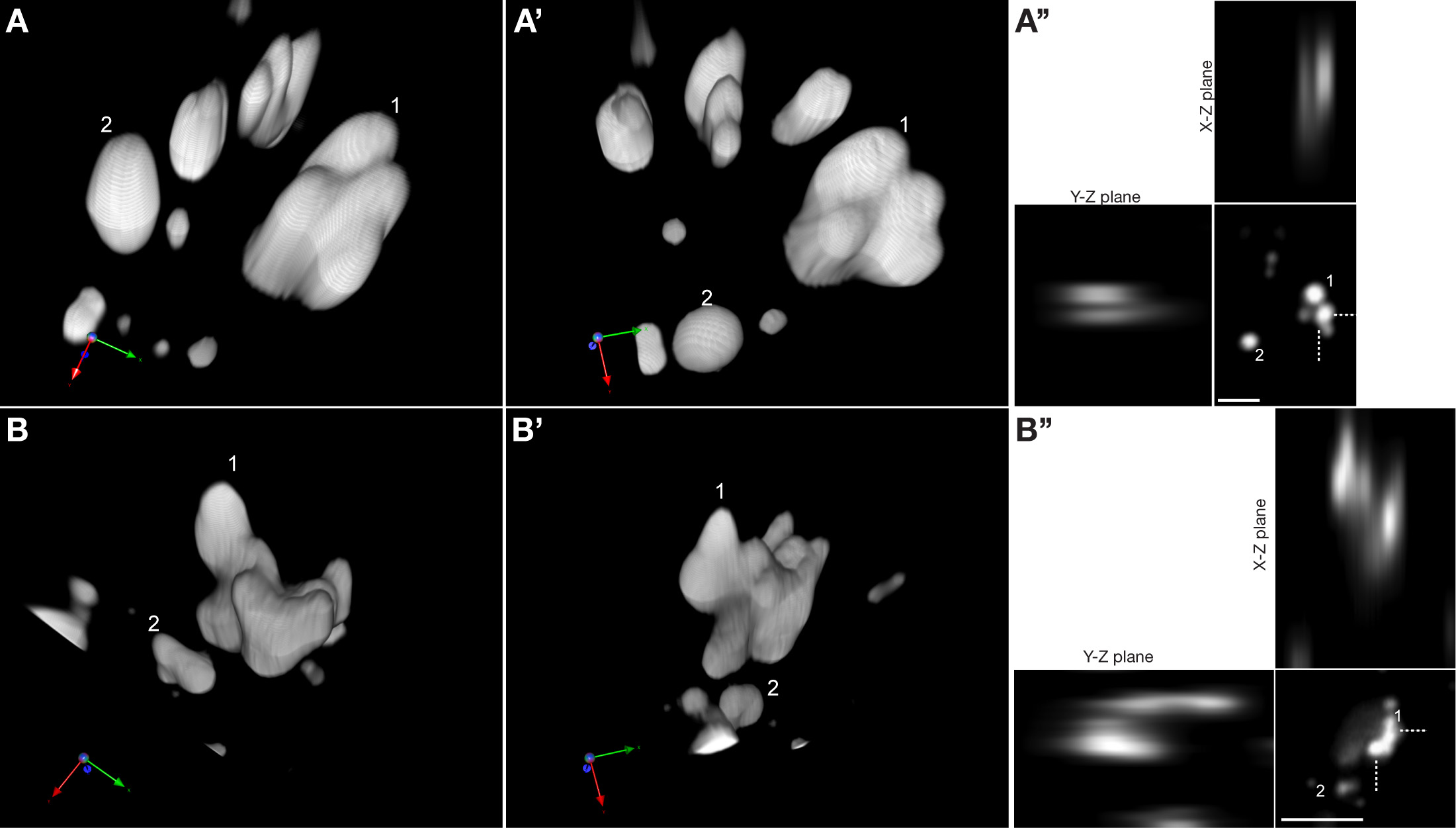

Supplement: Additional file 8 — LD aggregates stained with LipidTox. Two examples of LD aggregates from a live blastocyst stained with LipidTox and imaged by confocal microscopy. (A' and B') are different 3D views of the LD in (A and B) respectively. Corresponding LD are numbered in the two views, and the orientation is shown at bottom left. (A'' and B'') Optical section of the LD in (A and B) respectively, showing that LD are aggregates of smaller LD. LD numbers correspond to those in the volume renderings. Scale bar in (A'') = 2 μm and in (B'') = 5 μm. [file 1471-2121-11-38-S8.JPEG]
